# The Association Between Sarcopenia and Functional Improvement in Older and Younger Patients Who Completed Inpatient Rehabilitation: A Prospective Cohort Study

**DOI:** 10.3389/fresc.2021.692896

**Published:** 2021-10-21

**Authors:** Irina Churilov, Leonid Churilov, Kim Brock, David Murphy, Richard J. MacIsaac, Elif I. Ekinci

**Affiliations:** ^1^Department of Rehabilitation, St Vincent's Hospital Melbourne, Fitzroy, VIC, Australia; ^2^Department of Medicine, Austin Health, Melbourne Medical School, The University of Melbourne, Heidelberg, VIC, Australia; ^3^Melbourne Brain Centre at Royal Melbourne Hospital, Melbourne Medical School, The University of Melbourne, Parkville, VIC, Australia; ^4^Department of Physiotherapy, St Vincent's Hospital Melbourne, Fitzroy, VIC, Australia; ^5^Department of Endocrinology and Diabetes, St Vincent's Hospital Melbourne, Fitzroy, VIC, Australia; ^6^Department of Endocrinology, Austin Health, Heidelberg, VIC, Australia

**Keywords:** sarcopenia, rehabilitation, inpatient rehabilitation, functional improvement, muscle

## Abstract

**Objective:** To investigate the association between sarcopenia and functional improvement in patients older and younger than 65 years upon completion of an inpatient rehabilitation program.

**Design:** Prospective cohort study.

**Participants:** Adult consecutive patients who completed the inpatient rehabilitation program at a metropolitan tertiary referral hospital general inpatient rehabilitation unit.

**Methods:** Sarcopenia status was determined using the European Working Group on Sarcopenia in Older People 2 algorithm, using muscle mass measured by BioImpedance Analysis and grip strength. Progress in rehabilitation was measured using change in the Functional Independence Measure and Goal Attainment Scaling score. To investigate the age group by sarcopenia status interaction we used quantile regression models with bootstrapped standard error estimation for functional improvement and linear regression model with robust standard error estimation for GAS score.

**Results:** 257 participants [128 (50%) male, median age 63 years (IQR: 52–72)], 33(13%) with sarcopenia, completed inpatient rehabilitation [median length of stay 16 days (IQR: 11–27.5)]. Participants' median Functional Independence Measure change was 24 (IQR 15–33.5) and mean total Goal Attainment Scaling score was 57.6 (SD 10.2). Adjusting for admission Functional Independence Measure score, the median difference in Functional Independence Measure change between participants with and without sarcopenia was: −4.3 (95% CI: −10.6, 1.9); *p* = 0.17 in participants 65 years and younger, and 4.6 (95% CI: 1.0, 8.2); *p* = 0.01 in participants older than 65; age-by-sarcopenia interaction *p* = 0.02.

**Conclusions:** Unlike younger people, older people with sarcopenia have greater functional improvement in inpatient rehabilitation than those without sarcopenia.

## Introduction

Sarcopenia is a disorder of muscle mass and function that is associated with disability, morbidity and mortality (1). Sarcopenia in people in the community is associated with increased risk of falls (2), greater functional disability (3), loss of independence (1), and cognitive impairment (4). Sarcopenia has been a focus of significant research effort recently, with the number of publications on sarcopenia in PubMed growing exponentially since 1993 (5). In late 2016, sarcopenia was recognized as an independent medical condition by the International Classification of Diseases, Tenth Revision, Clinical Classification (ICD-10-CM) code, emphasizing its clinical significance.

While older people who have sarcopenia are known to have an increased length of stay and higher mortality when admitted to acute hospitals compared to those without sarcopenia (6), there is conflicting and limited evidence as to whether the presence of sarcopenia is associated with poorer outcomes in people admitted to subacute inpatient geriatric rehabilitation (7, 8). In the population of 127 participants over 70 years of age, Landi et al. (7) found that patients with sarcopenia admitted to rehabilitation after a hip fracture had similar function at the time of admission but made less functional gains by discharge. Sanchez-Rodriguez et al. (8) reported that in the population of 99 participants over 75 years of age, patients with sarcopenia had worse function on admission and on discharge than patients without sarcopenia, but there was no difference in change of function between these two groups.

Despite sarcopenia being prevalent in people both older and younger than 65 years in inpatient rehabilitation (9), the association between the presence of sarcopenia and rehabilitation outcomes in general inpatient rehabilitation, without excluding specific age or diagnostic groups, has not, to our knowledge, been investigated to date. The objectives of this study were therefore to investigate the association between sarcopenia status and improvement in function in patients older and younger than 65 years upon completion of inpatient rehabilitation program, measured by Functional Independence Measure (FIM) change and Goal Attainment Scaling (GAS) score. We hypothesized that the strength of this association differs between patients older and younger than 65 years.

## Materials and Methods

This study is reported in accordance with Strengthening the Reporting of Observational Studies in Epidemiology guidelines for reporting of cohort studies (10). This is a prospective cohort study.

### Settings and Participants

Consecutive patients who were admitted to the general inpatient rehabilitation unit at St Vincent's Hospital Melbourne, a metropolitan tertiary referral hospital, between November 2016 and March 2020, were assessed for eligibility to participate in this prospective cohort study. This rehabilitation unit accepts patients in the broad diagnostic groups of neurological impairments (including stroke, brain cancer, demyelinating diseases), musculoskeletal impairments (including arthroplasty, fractures, cancer surgery), spinal cord injury (including traumatic and non-traumatic etiologies), cardiac impairments (including cardiac failure, acute myocardial infarction, cardiac surgery), amputations, deconditioning, and other diagnoses. Adult patients who were able to provide informed consent, had an expected length of stay of more than 5 days, and were able to undergo BioImpedance Analysis testing (patients who did not have cardiac pacemakers, other implantable electronic devices, or amputations above ankle or wrist) were eligible to participate.

The rehabilitation programs at St Vincent's Hospital Melbourne rehabilitation unit are individually designed for each patient, taking into account their diagnostic group, impairments, level of function and rehabilitation goals. Allied health inputs available include physiotherapy, occupational therapy, speech pathology, dietetics, and social work.

Participants who did not complete their inpatient rehabilitation program, in that they continued to require inpatient care on discharge from the unit, were subsequently excluded from the final analysis as the objective of the study was to investigate the association between sarcopenia status and rehabilitation outcomes upon completion of inpatient rehabilitation program. There were two reasons for failing to complete the rehabilitation program: a significant clinical deterioration that necessitated transfer to an acute facility, and transfer to a local inpatient rehabilitation provider for participants who have ongoing rehabilitation goals but are best managed at a local facility.

Written informed consent was obtained from all participants. The study was approved by the St Vincent's Hospital Melbourne Human Ethics Committee (approval number LRR/16/SVHM/160).

### Participant Assessment and Diagnosis of Sarcopenia

Information collected about the participants on admission to the Rehabilitation Unit included age, gender, premorbid accommodation, length of stay at the acute unit immediately preceding admission to rehabilitation, admission diagnoses, height, weight, grip strength, muscle mass as measured by BioImpedace Analysis, Charlson Comorbidity Index, and admission Functional Independence Measure. Information collected about the participants on discharge included length of stay at the rehabilitation unit, discharge destination, and discharge Functional Independence Measure score.

Grip strength was measured using Jamar Hydraulic Hand Dynamometer (Lafayette Instrument Company, USA) (11). Two measurements were taken with the participant's dominant hand, unless that hand was affected by a unilateral condition such as stroke or amputation. The higher value of the two measurements was used for the analysis.

Muscle mass was measured using a commercially available ImpediMed SFB7 device (ImpediMed Limited, CA, USA) which determines the electrical impedance of the patient's body, which can subsequently be used to estimate the lean body mass and body fat. BioImpedance Analysis is contraindicated in patients who have a permanent pacemaker or another implanted electronic device, and its accuracy is not established in people with limb amputations above ankle or above wrist. Muscle mass was estimated from the resistance (R) and reactance (Xc) measured at 50 kHz according to the equation developed by Sergi et al. (12):

$$\begin{array}{l} \text{Musclemass}=-3.964+0.227\;*\text{ height}2/\text{R}+0.095\;*\text{ weight}\\ +1.384*\text{gender}+0.064\;*\text{ Xc}, \end{array}$$

where muscle mass is in kilograms, height is in centimeters, R is in ohms, age is in years, and gender is 0 for women and 1 for men.

Sarcopenia status of the participants was determined using the algorithm and cut off values recommended by the European Working Group on Sarcopenia in Older People 2 (EWGSOP2) (13). This algorithm determines the participants' sarcopenia status by measuring their grip strength and muscle mass, and sarcopenia is considered to be present if both measurements are low. The cut off values used for grip strength are 16 kg for women and 27 kg for men, and the cut off values used for skeletal mass index (SMI, muscle mass/height 2) are 7 kg/m2 for men and 5.5 kg/m2 for women.

Charlson Comorbidity Index is an instrument that measures the individual's burden of disease as determined by comorbidities. This index was originally developed to predict 1 year mortality (14), but is more commonly used to describe the extent of an individual's illness and disability (15).

Functional Independence Measure (FIM) tool (16) is a 126-point score that assesses the participant's independence in the domains of self-care, continence, transfer, mobility, communication, and cognition. It has been validated as having high internal consistency and adequate discriminative capabilities in inpatient rehabilitation patients (17), and is one of the most commonly used functional measures in rehabilitation (18). In addition to the admission and discharge FIM score, we also investigated FIM change, which is the difference between admission and discharge FIM, and FIM efficiency, calculated as the rate of FIM change per day of inpatient rehabilitation episode.

Goal Attainment Scaling (GAS) is a method of measuring the degree of goal achievement that is completed by treating therapists in consultation with the participants (19, 20), and involves several steps. At the beginning of admission, therapists trained in the GAS goal setting process supported participants in identifying the level of performance they hoped to achieve by the time of discharge from the inpatient rehabilitation unit. The therapists used these aspirations to formulate a number of goals that were specific and measurable. Five goal achievement levels were formulated, namely the expected level of achievement and two levels above and below the expected level. Participants nominated the degree of importance of the goals, while the therapists ranked the goals' degree of difficulty. On discharge from rehabilitation, the degree of goal achievement was assessed by treating therapists. The GAS score was calculated using the equation

$$\begin{array}{l} T=50+\frac{10\Sigma w_{i}x_{i}}{\sqrt{0.7\Sigma w_{i}^{2}+0.3{\left(\Sigma w_{i}\right)}^{2}}} \end{array}$$

where i is the number of the individual goal, wi = weight assigned to the i-th goal (importance multiplied by difficulty) and xi = the score of the i-th goal (21).

### Sample Size Estimation

Power analysis was conducted using GPower software (22). Based on the audit conducted at St Vincent's Hospital Melbourne inpatient rehabilitation unit, the anticipated prevalence of sarcopenia in inpatient rehabilitation was expected to be in the range of 15–20%. This was subsequently confirmed in Churilov et al. (9). Thus, the ratio of 1 participant with sarcopenia to 4–5 participants without sarcopenia was assumed for this power analysis.

A total sample size of 240 participants was found to yield 80% power to detect the difference in the individual outcome measures of interest between participants with and without sarcopenia that would correspond to medium effect (Cohen's *d* = 0.5), assuming alpha = 0.05.

Considering the uncertainties in the underlying sarcopenia prevalence and potential exclusion due to failure to complete rehabilitation, the sample size for this study was conservatively estimated as 300 participants.

### Statistical Analysis

To investigate the potential of selection bias, participant characteristics on admission to rehabilitation were compared between those included and excluded from the analysis due to failure to complete rehabilitation using Wilcoxon-Mann-Whitney test for continuous variables and Chi square or Fisher's exact test as appropriate for categorical variables.

Participant characteristics on admission to rehabilitation in 65 years and younger and older than 65 years groups, as well as those for participants with and without sarcopenia, were summarized as medians (interquartile ranges) for continuous characteristics and as counts (proportions) for categorical characteristics, and were compared using Wilcoxon-Mann-Whitney Ranksum test or Chi-square (Fisher's exact) test as appropriate.

To investigate the age group by sarcopenia status interaction for functional improvement, quantile regression models with bootstrapped standard error estimation were used. Included independent variables were age (dichotomized as 65 years old and younger, and older than 65 years), sarcopenia status, age group-by-sarcopenia multiplicative interaction term, and FIM on admission, while individual dependent variables in separate models were change in total FIM score and change in Motor FIM score. Quantile regression estimates differences in the quantiles of the specific functional improvement measure between participants in two different groups. Standard assessment of collinearity was conducted using variance inflation factors (VIF) and condition number. For both of the investigated functional improvement measures, we report the magnitudes of baseline FIM-adjusted 25th, median and 75th percentile differences (95% CIs) between participants with and without sarcopenia in younger and older participant groups, as well as the respective *p*-value for interaction. A significant *p*-value for interaction is indicative of statistically significant difference between older and younger participants in strength of association between sarcopenia status and functional improvement in inpatient rehabilitation.

To investigate the age group by sarcopenia status interaction for GAS score, a linear regression model with robust standard error estimation with age (dichotomized as 65 years old and younger, and older than 65 years), sarcopenia status, age group-by-sarcopenia multiplicative interaction term as independent variables and GAS score as dependent variable was used. GAS scores are designed to follow normal distribution (23) and GAS goal setting process is meant to naturally take potential confounders such as age, comorbidity burden and function on admission into consideration, hence we used unadjusted linear regression model with robust standard error estimation. We report the magnitudes of mean differences (95% CIs) between participants with and without sarcopenia in younger and older participant groups, as well as the respective *p*-value for interaction. A significant *p*-value (*p* < 0.05) for interaction is indicative of statistically significant difference between older and younger participants in strength of association between sarcopenia status and GAS score in inpatient rehabilitation.

Statistical analysis was conducted using Stata 15IC statistical software (StataCorp, College Station, TX, USA). Two sided *p*-values of <0.05 were regarded as indicative of statistical significance. No adjustment was made for the multiplicity of statistical testing.

## Results

### Participants' Characteristics

**Figure 1:**
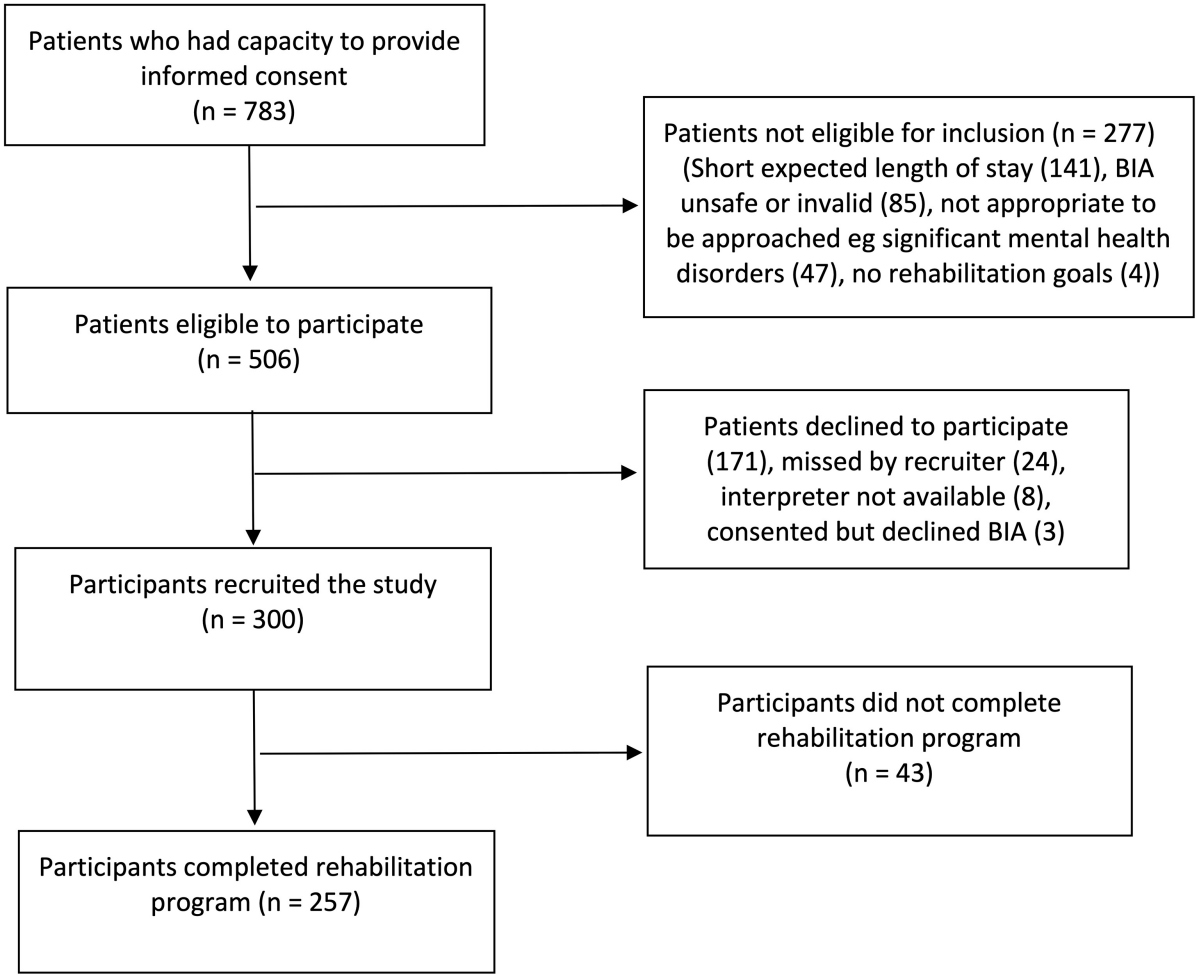
Study recruitment flow chart (BIA, BioImpedance analysis).

Three hundred participants were recruited to the study. Of these, 43 (14%) participants did not complete their inpatient rehabilitation program, as they continued to require inpatient care on discharge from the unit. Reasons for failing to complete the rehabilitation program were transferring to a local rehabilitation facility (26 participants, 60%) and transferring to acute hospital and not returning to rehabilitation (17 participants, 40%). The remaining 257 participants were included in final analysis. The study recruitment flow is summarized in Figure 1.

Overall, compared to the recruited participants who were excluded from the analysis, participants included in the analysis had lower Charlson Comorbidity Index [included: median 1 (IQR 0–2) vs. excluded: median 2 (IQR1–5), *p* < 0.001] and acute length of stay [9 days (IQR 5–18) vs. 16 days (IQR 9–28), *p* < 0.001], and higher total admission FIM [91 (IQR 77–99) vs. 77 (IQR 66–92), *p* = <0.001]. There were also fewer participants with low SMI (included: 23% vs. excluded: 47%, *p* = 0.003) and positive sarcopenia status (13 vs. 26%, *p* = 0.04) included in the study. For detailed description of included and excluded participants see Table 1 Online Appendix.

**Table 1:** Admission characteristics of participants older and younger than 65 years stratified by sarcopenia status.

	**Participants 65 years old and younger**				**Participants older than 65 years**				*p* ^*^
	**Overall, *n* = 137**	**No sarcopenia, *n* = 122**	**Sarcopenia, *n* = 15**	***p* ^∧^**	**Overall, *n* = 120**	**No sarcopenia, *n* = 102**	**Sarcopenia, *n* = 18**	***p* ^∧^**	
Age (years, median, IQR)	53 (42–60)	53 (40–60)	50 (46–56)	0.57	73 (69–78)	74 (69–79)	71 (69–72)	0.03	<0.001
Male gender (*n*, %)	71 (52)	61 (50)	10 (67)	0.28	57 (48)	43 (42)	14 (78)	0.009	0.53
Length of stay in acute hospital (days, median, IQR)	10 (4–20)	10.5 (4–20)	7 (4–26)	0.93	9 (5–17)	8 (5–16)	15 (9–31)	0.016	0.94
Charlson score, not age adjusted (median, IQR)	0 (0–2)	0 (0–2)	1 (0–3)	0.29	1 (0–2)	1 (0–2)	1 (0–2)	0.72	0.21
Body mass index [weight (kg)/height (cm)^∧^2], median, IQR	28.2 (24.1–34.1)	28.8 (24.9–35.2)	21 (18.6–24.5)	<0.001	28.3 (23.4–33.9)	29.7 (26.2–34.8)	22.3 (18.3–22.9)	<0.001	0.91
Low grip strength (*n*, %)	37 (27)	22 (18)	15 (100)	<0.001	52 (43)	34 (33)	18 (100)	<0.001	0.008
Low muscle mass (*n*, %)	29 (21)	14 (11)	15 (100)	<0.001	31 (26)	13 (13)	18 (100)	<0.001	0.46
Admission FIM	91 (77–100)	90.5 (78–99)	93 (62–109)	0.84	89 (77–99)	89.5 (78–99)	85 (73–97)	0.57	0.55
Admission motor FIM	57 (45–66)	56.5 (46–65)	62 (28–74)	0.8	55 (43–64)	55.5 (46–64)	52 (38–62)	0.52	0.53

Out of 257 participants who completed rehabilitation and were included in the analysis, 128 (50%) were male. Participants' median age was 63 years (IQR 52–72), median height was 167 cm (IQR 160–175), and median weight was 80 kg (IQR 66–94 kg). Eighty nine participants (35%) had low grip strength. Their broad diagnostic groups were: neurological 55 (21%), musculoskeletal 94 (37%), spinal 34 (13%), cardiac 10 (4%), amputee 4 (2%), restorative/other 60 (23%). Participants' premorbid accommodation was: home alone 91 (35%), home with others 160 (62%), residential care 4 (2%), no fixed address 2 (1%). The discharge destinations of the participants were as follows: 74 (29%) home alone, 168 (65%) home with others, 15 (6%) supported accommodation.

Out of 257 participants who completed rehabilitation, 33 (13%, 95% CI: 9%, 18%) had sarcopenia. No statistically significant difference was observed in the prevalence of sarcopenia between participants 65 years and younger [15/137 (11%)], and older than 65 years [18/120 (15%), *p* = 0.36]. The admission characteristics of participants older and younger than 65 years, as well as admission characteristics stratified by sarcopenia status are shown in Table 1.

Participants' median length of stay in inpatient rehabilitation was 16 days (IQR 11–27.5). In participants 65 years or younger, length of stay was: in participants without sarcopenia (median 16.5 days, IQR 11–27) and those with sarcopenia (median 14 days, IQR 10–25), admission FIM adjusted *p* = 0.43. In participants older than 65 years length of stay in rehabilitation was: in participants without sarcopenia (median 15 days, IQR 10–25), and those with sarcopenia (median 20 days, IQR 11–31), admission FIM adjusted *p* = 0.23.

### Functional Outcomes: FIM Change

Participants' median total discharge FIM was 116 (IQR 111–120). The total median discharge FIM in participants aged 65 and younger was 116 (IQR 108–120), and the median in participants older than 65 was 117 (IQR 112–120).

**Figure 2:**
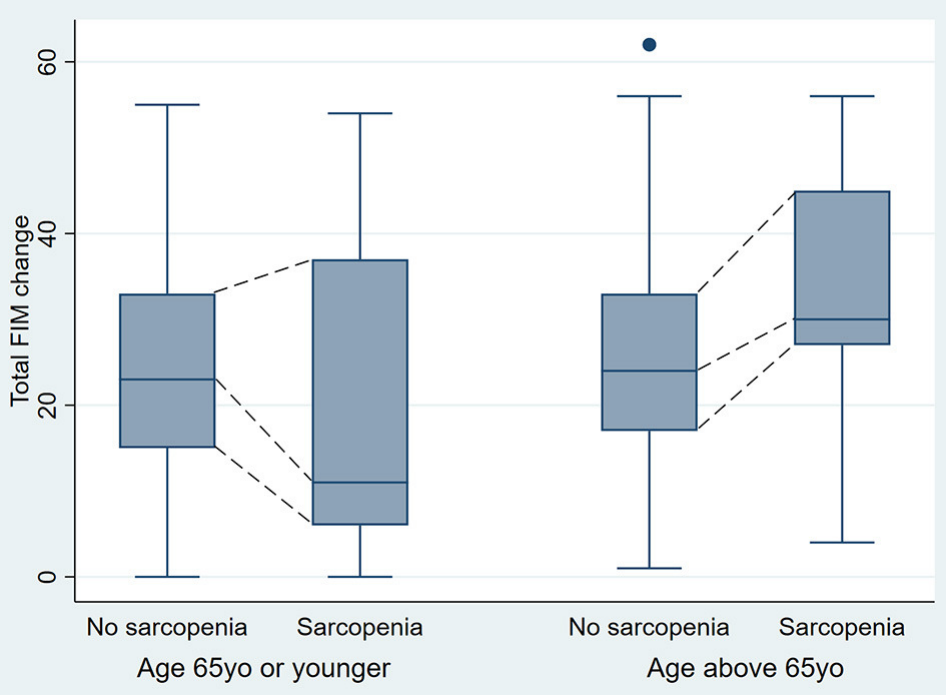
Total FIM change in younger and older participants by sarcopenia status (One outlier value was removed from the Figure for ease of interpretation, but was still included in the analysis).

Participants' median FIM change was 24 (IQR 15–33.5). Statistically significant differences between participants aged 65 and younger and participants older than 65 in the strength of association between sarcopenia status and total FIM change (significant age-by-sarcopenia interaction, *p* = 0.02) were observed for the median percentile. Adjusting for admission FIM score, in participants 65 years and younger the median difference in FIM change between participants with and without sarcopenia was −4.3 (95% CI −10.6, 1.9); *p* = 0.17, while in participants older than 65 this difference was 4.6 (95% CI 1.0, 8.2); *p* = 0.01 (Figure 2). The values for 25 and 75th percentiles are shown in Table 2.

**Table 2:** Participants' total functional independence measure (FIM) change, motor FIM change, and Goal Attainment Scaling scores of participants older and younger than 65 years stratified by sarcopenia status.

	**Participants 65 years and younger**, ***n*** **= 137**				**Participants older than 65 years**, ***n*** **= 120**				***p* value for interaction**
	**No sarcopenia, *n* = 122**	**Sarcopenia, *n* = 15**	**Difference** **(95% CI)** ***p***		**No sarcopenia, *n* = 102**	**Sarcopenia, *n* = 18**	**Difference** **(95% CI)** ***p***		
Change in total functional independence measure (median, IQR)	23 (15, 33)	11 (6, 37)	25th percentile^*^	−5.4 (−24.7, 14.0) 0.58	23.5 (16, 33)	30 (27, 45)	25th percentile^*^	3.3 (−2.7, 9.2) 0.29	0.34
			Median^*^	−4.3 (−10.6, 1.9) 0.17			Median^*^	4.6 (1.0, 8.2) 0.01	0.02
			75th percentile^*^	−4 (−9.5, 1.5) 0.16			75th percentile^*^	1.5 (−1.7, 4.7) 0.35	0.04
Change in motor functional independence measure (median, IQR)	23 (14, 33)	11 (6, 37)	25th percentile^*^	−5.2 (−25.2, 14.8) 0.6	23.5 (16, 33)	28 (27, 45)	25th percentile^*^	4.4 (−1.6, 10.4) 0.15	0.35
			Median^*^	−3.6 (−8.6, 1.4) 0.16			Median^*^	3.1 (−0.5, 6.8) 0.09	0.04
			75th percentile^*^	−3.9 (−8.9, 1.0) 0.12			75th percentile^*^	2.2 (−1.1, 5.5) 0.2	0.04
GAS score (mean, SD)	58.3 (10.5)	55.6 (10.1)	Mean	−2.8 (−8.2, 2.6) 0.31	56.7 (10.3)	59.0 (7.2)	Mean	2.3 (−1.6, 6.2) 0.25	0.13

* *Difference adjusted for admission FIM*.

### Functional Outcomes: Motor FIM Change

Participants' median motor discharge FIM was 82 (IQR 76–86). The total median discharge FIM in participants aged 65 and younger was 82 (IQR 75–86), and the median in participants older than 65 was 82 (IQR 77–86).

**Figure 3:**
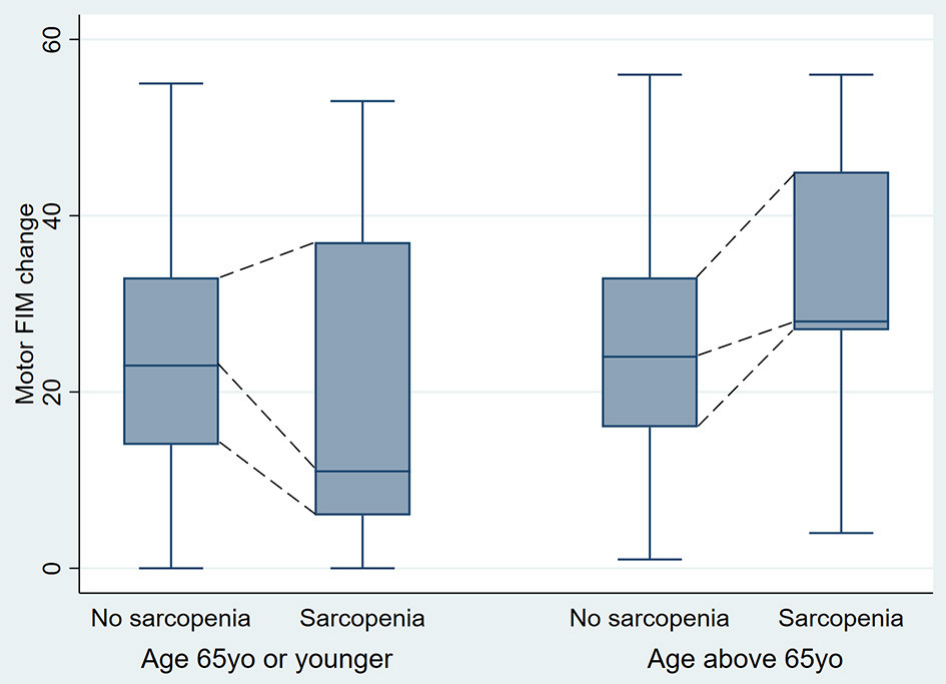
Motor FIM change in younger and older participants by sarcopenia status (One outlier value was removed from the Figure for ease of interpretation, but was still included in the analysis).

Participants' median motor FIM change was 24 (IQR 15–33). Statistically significant differences between participants aged 65 and younger and participants older than 65 in the strength of association between sarcopenia status and motor FIM change (significant age-by-sarcopenia interaction, *p* = 0.04) were observed for the median percentile. Adjusting for admission FIM score, in participants 65 years and younger the median difference in motor FIM change between participants with and without sarcopenia was −3.6 (95% CI −8.6, 1.4); *p* = 0.16, while in participants older than 65 such a difference was 3.1 (95% CI −0.5, 6.8); *p* = 0.09 (Figure 3). The values for 25th and 75th percentiles are shown in Table 2.

### Functional Outcomes: GAS Score

Seven participants (2.7%) had missing GAS scores. Participants' mean total GAS score was 57.6 (SD 10.2). The total mean GAS score in participants aged 65 and younger was 58.0 (SD 10.4), and the mean in participants older than 65 was 57.0 (SD 9.9).

No evidence of statistically significant differences between participants aged 65 and younger and participants older than 65 in the strength of association between sarcopenia status and GAS score was observed (age-by-sarcopenia interaction, *p* = 0.13). In participants 65 years and younger the mean difference in GAS score between participants with and without sarcopenia was −2.8 (95% CI −8.2, 2.6); *p* = 0.31, while in participants older than 65 this difference was 2.3 (−1.6, 6.2); *p* = 0.25.

## Discussion

This study demonstrated that there was a statistically significant difference in the strength of association between sarcopenia status and functional improvement, as measured by total and motor FIM change, in participants older and younger than 65 years. The diagnosis of sarcopenia was associated with a greater median FIM change during admission to inpatient rehabilitation in participants older than 65 years, while there was no evidence of such association in participants 65 years and younger. This is, to our knowledge, the first study to compare the association between sarcopenia and functional improvement in older and younger participants.

The novel finding that the presence of sarcopenia is associated with greater functional improvement in older people was unexpected, because it was not consistent with two earlier studies, which reported that participants who had sarcopenia progressing either at the same rate (8) or worse (7) than participants without sarcopenia. However, the participant characteristics in these two studies differed to those observed in our study in that the participants in both Sanchez-Rodriguez et al. (8) and Landi et al. (7) studies were older [mean age 84.6 years (SD 6.6) and mean age 81.3 years “+/– 4.8 years” respectively] vs. median 63 years (IQR 52–72) in the present study. Also, while the participants in our study were recruited from a wide range of diagnostic groups, Sanchez-Rodriguez et al. (8) investigated participants with deconditioning, and Landi et al. (7) recruited participants following hip fractures.

Our study sample included 137/257 (53%) participants 65 years or younger. Despite higher than expected prevalence of sarcopenia in these participants, we identified no evidence of association between the presence of sarcopenia and FIM change during admission in this younger group. As this is the first study that examines functional improvement in younger people with sarcopenia and Type II error cannot be excluded, further investigations in this population may be warranted.

The observed sarcopenia-by-age group interaction for functional improvement indicates that the presence of advancing age potentially amplifies the association between sarcopenia and functional performance. A hypothetical explanation for our findings is that some participants in this study could have acute rather than chronic sarcopenia. Acute sarcopenia is defined by EWGSOP as lasting for <6 months, usually develops in response to an acute illness, and is often associated with hospitalization, developing in response to the combination of muscle disuse and acute inflammatory burden (12, 24). Acute sarcopenia is thought to be more easily treatable than chronic, while also being a risk factor for development of chronic sarcopenia (24) with associated increase in adverse health consequences. The possibility of the presence of acute sarcopenia in our cohort is further supported by the identified prevalence of sarcopenia on admission to rehabilitation being greater than that in the community. Since older people have less functional reserve (25), the development of sarcopenia may lead to a deterioration of their functional performance to a greater extent than in younger people. In older people with acute, potentially reversible, sarcopenia, increased physical activity, and dietary optimization that occur following admission to rehabilitation may be of greater functional benefit, thereby leading to a relatively greater improvement in functional performance. The way to further investigate this potential explanation of our findings, systematic screening for sarcopenia would be required on admission to acute hospital, to assist in differentiating between acute and chronic sarcopenia on admission to rehabilitation.

We found no evidence of difference in the strength of association between sarcopenia status and functional improvement as measured by GAS score in participants older and younger than 65 years, a finding consistent with the nature of GAS goal setting. GAS goals were set by experienced therapists who were involved in participants' clinical care. The therapists were taking into account participants' admission performance and perceived potential to improve, which would likely be affected by the participants' sarcopenia status.

This study has limitations. The setting of the study was a single rehabilitation health service; however, a wide range of rehabilitation diagnostic groups was included that is reflective of the range of diagnoses seen in inpatient rehabilitation. The participants who completed rehabilitation and were therefore included in the analysis had statistically significantly lower Charlson Comorbidity Index and acute length of stay, higher admission FIM and lower prevalence of sarcopenia compared to those who did not complete rehabilitation. Therefore, our findings are applicable to participants who complete inpatient rehabilitation rather than those for whom inpatient care continues after discharge from the index admission. Further, we were not able to ascertain whether participants' sarcopenia was acute or chronic due to only performing a single assessment on admission to rehabilitation.

In conclusion, this study found that there was a statistically significant difference in the strength of association between sarcopenia status and functional improvement, as measured by total and motor FIM change, in participants older and younger than 65 years, as well as a significant association between sarcopenia and total FIM change in older than 65 years. Further investigation is needed to ascertain whether this association occurs with acute or chronic sarcopenia, and whether targeted sarcopenia treatment further improves outcomes in inpatient rehabilitation population.

## Data Availability Statement

The datasets presented in this article are not readily available because sharing of this data is not covered by the Ethics approval. Requests to access the datasets should be directed to Irina Churilov, irina.churilov@svha.org.au.

## Ethics Statement

The studies involving human participants were reviewed and approved by St Vincent's Hospital Melbourne. The patients/participants provided their written informed consent to participate in this study.

## Author Contributions

IC: conception and design, acquisition of data, analysis and interpretation of data, drafting the article, and final approval. LC: conception and design, analysis and interpretation of data, revising the article critically for important intellectual content, final approval. KB: conception and design, acquisition of data, revising the article critically for important intellectual content, final approval. DM: conception and design, revising the article critically for important intellectual content, final approval. RM and EE: conception and design, interpretation of data, revising the article critically for important intellectual content, final approval. All authors contributed to the article and approved the submitted version.

## Conflict of Interest

LC has received research funding from The National Health and Medical Research Council of Australia and Medical Research Future Fund of Australia for unrelated research. EE's institution receives research funding from Sanofi, Eli Lilly, Gilead, Novo Nordisk and Dimerix. EE has received research funding from The National Health and Medical Research Council of Australia, Sir Edward Weary Dunlop medical research foundation and The Juvenile Diabetes Research Foundation for unrelated research. RM has received research grants from Novo Nordisk, Servier, Medtronic, The Rebecca Cooper Medical Research Foundation, St Vincent's Research Foundation, The Juvenile Diabetes Research Foundation, Grey Innovations, The Diabetes Australia Research Trust/Program and The National Health and Medical Research Council of Australia. He has also has received honoraria for lectures from Eli Lilly, Novo Nordisk, Sanofi Aventis, Astra Zeneca, Merck Sharp & Dohme, Norvartis and Boehringer Ingelheim and is on the advisory boards for Novo Nordisk, Boehringer Ingelheim-Eli Lilly Diabetes Alliance and Astra Zeneca. Travel support has been supplied by Novo Nordisk, Sanofi and Boehringer Ingelheim. He has been a principal investigator for industry sponsored clinical trials run by Novo Nordisk, Bayer, Johnson-Cilag and Abbive. He is also a council member for the Australian Diabetes Society (ADS) but the views expressed in this manuscript should are his own and do not necessarily reflect those of the ASD. The remaining authors declare that the research was conducted in the absence of any commercial or financial relationships that could be construed as a potential conflict of interest.

## Publisher's Note

All claims expressed in this article are solely those of the authors and do not necessarily represent those of their affiliated organizations, or those of the publisher, the editors and the reviewers. Any product that may be evaluated in this article, or claim that may be made by its manufacturer, is not guaranteed or endorsed by the publisher.
