# Supplementary material for: The Association Between Sarcopenia and Functional Improvement in Older and Younger Patients Who Completed Inpatient Rehabilitation: A Prospective Cohort Study
Source: Front Rehabil Sci. 2021 Oct 21;2:692896. doi: 10.3389/fresc.2021.692896 (PMC9397850; doi:10.3389/fresc.2021.692896)
Supplement: Supplementary file 1 [file Table_1.DOCX]

Supplementary Material

**
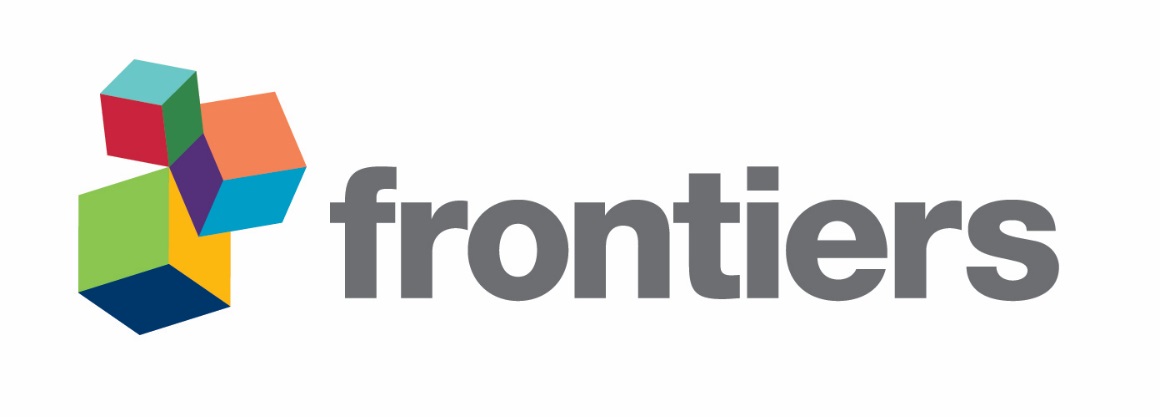
**

**Supplementary Table I.** Included vs excluded participants’ characteristics

|  | Excluded (n=43) | Included (n=257) | p value |
| --- | --- | --- | --- |
| Age (years, median, IQR) | 66 (61-74) | 63 (52-72) | 0.06 |
| Male gender (number, %) | 27 (63) | 128 (50) | 0.14 |
| LOS in acute (days, median, IQR) | 16 (9-28) | 9 (5-18) | <0.001 |
| Charlson comorbidity index (median, IQR) | 2 (1-5) | 1 (0-2) | <0.001 |
| Height (cm, median, IQR) | 168 (163-176) | 167 (160-175) | 0.32 |
| Weight (kg, median, IQR) | 78.5 (71-86.9) | 79.6 (65.7-94.1) | 0.85 |
| Total admission FIM (median, IQR) | 77 (66-92) | 90.5 (77-99) | <0.001 |
| Low grip strength (number, %) | 20 (47) | 89 (35) | 0.17 |
| Low Skeletal Muscle Index (number, %) | 20 (47) | 60 (23) | 0.003 |
| Positive sarcopenia status (number, %) | 11 (26) | 33 (13) | 0.04 |
